# Supplementary material for: How do supervisor-student relationship affect anxiety of graduate students? ——The mediating role of research self-efficacy and the moderating role of mindset
Source: PLoS One. 2025 Aug 20;20(8):e0328068. doi: 10.1371/journal.pone.0328068 (PMC12367134; doi:10.1371/journal.pone.0328068)
Supplement: Attachment — Submitted filename: Reviewer Comments.docx [file pone.0328068.s002.docx]

Journal Requirements:

1. **When submitting your revision, we need you to address these additional requirements.**

**Please ensure that your manuscript meets PLOS ONE's style requirements, including those for file naming. The PLOS ONE style templates can be found at** [**https://journals.plos.org/plosone/s/file?id=wjVg/PLOSOne_formatting_sample_main_body.pdf**](https://urldefense.proofpoint.com/v2/url?u=https-3A__journals.plos.org_plosone_s_file-3Fid-3DwjVg_PLOSOne-5Fformatting-5Fsample-5Fmain-5Fbody.pdf&d=DwQGaQ&c=slrrB7dE8n7gBJbeO0g-IQ&r=7lapq_ae5nufOEz9sHrMRw&m=Nx6xT2p3gZDTVahmQ7MNoLiV1sREeWznn_og_tbwJCvLDqdaHBxgbjsJw3RzQRcK&s=GBm-EszBOmjGZ1Mwhm90dOV8r1imyd4hyhCrqj0zfWE&e=) **and** [**https://journals.plos.org/plosone/s/file?id=ba62/PLOSOne_formatting_sample_title_authors_affiliations.pdf**](https://urldefense.proofpoint.com/v2/url?u=https-3A__track.editorialmanager.com_CL0_https-3A-252F-252Fjournals.plos.org-252Fplosone-252Fs-252Ffile-253Fid-3Dba62-252FPLOSOne-5Fformatting-5Fsample-5Ftitle-5Fauthors-5Faffiliations.pdf_1_010f0195b9988848-2D966fb460-2D2170-2D4ca3-2D9ad4-2D3374b9636348-2D000000_TEOwpK-5FbuWYY-5FxdcPBUmfsi6ebjvCTnILRj4tELyt4Q-3D203&d=DwMGaQ&c=slrrB7dE8n7gBJbeO0g-IQ&r=7lapq_ae5nufOEz9sHrMRw&m=Nx6xT2p3gZDTVahmQ7MNoLiV1sREeWznn_og_tbwJCvLDqdaHBxgbjsJw3RzQRcK&s=S00EoLWE2mgMZ0RhTFv6qdHtBhPV-rj5WBzLfvjr93Y&e=)

Thank you for your comments. We have now modified the format of the manuscript to meet the requirement of the journal.

2. **We note that you have indicated that there are restrictions to data sharing for this study. For studies involving human research participant data or other sensitive data, we encourage authors to share de-identified or anonymized data. However, when data cannot be publicly shared for ethical reasons, we allow authors to make their data sets available upon request. For information on unacceptable data access restrictions, please see** [**http://journals.plos.org/plosone/s/data-availability#loc-unacceptable-data-access-restrictions**](https://urldefense.proofpoint.com/v2/url?u=http-3A__journals.plos.org_plosone_s_data-2Davailability-23loc-2Dunacceptable-2Ddata-2Daccess-2Drestrictions&d=DwQGaQ&c=slrrB7dE8n7gBJbeO0g-IQ&r=7lapq_ae5nufOEz9sHrMRw&m=Nx6xT2p3gZDTVahmQ7MNoLiV1sREeWznn_og_tbwJCvLDqdaHBxgbjsJw3RzQRcK&s=TVnUuWRUAw1s0LDjnKnHzLY2YY2SOYx2lj1IsjLq_kI&e=)**.**

Thank you for your reminder and suggestion. We reapplied to the Ethics Committee regarding the data request. We now make the data used in this study publicly available, posting it to the Open Science Framework (OSF) platform and creating anonymized links for reviewers to review. We also uploaded the questionnaire used in this study for reference.

https://osf.io/sgp5t/?view_only=27570214b73d4690b4f47d8311c7ea71

Before we proceed with your manuscript, please address the following prompts:

a) If there are ethical or legal restrictions on sharing a de-identified data set, please explain them in detail (e.g., data contain potentially identifying or sensitive patient information, data are owned by a third-party organization, etc.) and who has imposed them (e.g., a Research Ethics Committee or Institutional Review Board, etc.). Please also provide contact information for a data access committee, ethics committee, or other institutional body to which data requests may be sent.

b) If there are no restrictions, please upload the minimal anonymized data set necessary to replicate your study findings to a stable, public repository and provide us with the relevant URLs, DOIs, or accession numbers. Please see [http://www.bmj.com/content/340/bmj.c181.long](https://urldefense.proofpoint.com/v2/url?u=http-3A__www.bmj.com_content_340_bmj.c181.long&d=DwQGaQ&c=slrrB7dE8n7gBJbeO0g-IQ&r=7lapq_ae5nufOEz9sHrMRw&m=Nx6xT2p3gZDTVahmQ7MNoLiV1sREeWznn_og_tbwJCvLDqdaHBxgbjsJw3RzQRcK&s=uUfqN9zV0g4eL_-_LAymxNsChykNFPbyRh0vCOuJUQU&e=) for guidelines on how to de-identify and prepare clinical data for publication. For a list of recommended repositories, please see [https://journals.plos.org/plosone/s/recommended-repositories](https://urldefense.proofpoint.com/v2/url?u=https-3A__journals.plos.org_plosone_s_recommended-2Drepositories&d=DwQGaQ&c=slrrB7dE8n7gBJbeO0g-IQ&r=7lapq_ae5nufOEz9sHrMRw&m=Nx6xT2p3gZDTVahmQ7MNoLiV1sREeWznn_og_tbwJCvLDqdaHBxgbjsJw3RzQRcK&s=LDb0TF5up6UJ7rWc1WO9b3FdqFDl1ibVNtGyvPnjg8I&e=). You also have the option of uploading the data as Supporting Information files, but we would recommend depositing data directly to a data repository if possible.

Please update your Data Availability statement in the submission form accordingly.

Additional Editor Comments:
**The manuscript has a good deal of merit for publication after major revisions. The recommended citation is optional and should only be taken as a suggestion for the improvement of the manuscript.** 
Thank you for your comments. We carefully read the recommended citation and decided to cite that in the appropriate place. We hope this will enhance the synthesis of this study.

[Note: HTML markup is below. Please do not edit.]

Reviewers' comments:

Reviewer's Responses to Questions

**Comments to the Author**

1. Is the manuscript technically sound, and do the data support the conclusions?

The manuscript must describe a technically sound piece of scientific research with data that supports the conclusions. Experiments must have been conducted rigorously, with appropriate controls, replication, and sample sizes. The conclusions must be drawn appropriately based on the data presented.

Reviewer #1: Partly

2. Has the statistical analysis been performed appropriately and rigorously?

Reviewer #1: Yes

3. Have the authors made all data underlying the findings in their manuscript fully available?

The [PLOS Data policy](https://urldefense.proofpoint.com/v2/url?u=http-3A__track.editorialmanager.com_CL0_http-3A-252F-252Fwww.plosone.org-252Fstatic-252Fpolicies.action-2523sharing_1_010f0195b9988848-2D966fb460-2D2170-2D4ca3-2D9ad4-2D3374b9636348-2D000000_-5FZjfqEvEfpYGvjes12MMNfj-2Dv2GeC-2D4Z-2DdqUiEYvp6A-3D203&d=DwMGaQ&c=slrrB7dE8n7gBJbeO0g-IQ&r=7lapq_ae5nufOEz9sHrMRw&m=Nx6xT2p3gZDTVahmQ7MNoLiV1sREeWznn_og_tbwJCvLDqdaHBxgbjsJw3RzQRcK&s=DGEKbCUhnl0PLbqPa7_5n59ycMtXPXHDBbrPnmr-thQ&e=) requires authors to make all data underlying the findings described in their manuscript fully available without restriction, with rare exception (please refer to the Data Availability Statement in the manuscript PDF file). The data should be provided as part of the manuscript or its supporting information, or deposited to a public repository. For example, in addition to summary statistics, the data points behind means, medians and variance measures should be available. If there are restrictions on publicly sharing data—e.g. participant privacy or use of data from a third party—those must be specified.

Reviewer #1: No

4. Is the manuscript presented in an intelligible fashion and written in standard English?

PLOS ONE does not copyedit accepted manuscripts, so the language in submitted articles must be clear, correct, and unambiguous. Any typographical or grammatical errors should be corrected at revision, so please note any specific errors here.

Reviewer #1: Yes

5. Review Comments to the Author

Please use the space provided to explain your answers to the questions above. You may also include additional comments for the author, including concerns about dual publication, research ethics, or publication ethics. (Please upload your review as an attachment if it exceeds 20,000 characters)

Reviewer #1: I am pleased to have the opportunity to review your manuscript. The research topic is meaningful, but the manuscript currently has numerous issues that need to be addressed. In its current state, it is not sufficient for publication in this journal. Below are my detailed comments and suggestions for improvement:

Abstract
**Q1: The abstract lacks a clear statement of the research gap.**

Thank you for your comment. Our previous abstract did not clearly explain the gap between this study and previous studies. We have now added this to the objectives section. Due to the length limit for the abstract section, we will provide a more detailed introduction to the research gap between this study and previous studies in the introduction.

"Self-efficacy and mindset have been found to moderate the effect of negative life events on individuals' anxiety and depression levels. However, few studies have extended this conclusion to postgraduate student samples. "

**Q2: The conclusion should include both theoretical and practical implications of all findings (e.g., the significance of Research Self-efficacy).**

Thank you for your suggestion. We have now added a conclusion to the abstract, emphasizing the importance of the study's results for the promotion of the theory of self-determination in the field of education.

"In addition, this study extends the application of self-determination theory to the field of education by verifying the mediating role of research self-efficacy."

**Q3: In conclusion, the author expanded the scope of the research results. Anxiety is only one aspect of mental health, and this study is a cross-sectional quantitative study. The measurement of "anxiety" and "mental health" differs in scope and tools. Therefore, the conclusions should be carefully revised to align with the specific dependent variable (anxiety) studied.**

Thank you for your suggestion, and we have unified the point you have pointed out. It is inappropriate to generalize the results of anxiety and depression to mental health. Therefore, we have replaced “mental health” in the summary with the more accurate “anxiety and depression” to avoid confusion.

Introduction
**Q4: While the authors clearly explain why this study was conducted, the introduction lacks an analysis of the research gap and the study’s innovation.**

Thank you for your suggestion, and we have made further revisions to the introduction. We cite several research articles published within the past five years and compare the research gap between this study and these studies, including poor generalizability due to the sample scope and insufficient exploration of moderating effects.

**Q5: Before discussing the relationships between variables, the author lacks the reason and basis for studying the mediator and moderator. Specifically, what is the necessity of studying "Research Self-efficacy" and "Mindset"?**

Thank you for your comment. The reason we chose to study the Research Self-efficacy variable was that previous studies have found that Research Self-efficacy has a mediating effect in this topic, but the sample size limited the generalizability of the previous research. At the same time, Mindset has been found to significantly moderate depressive symptoms caused by subjective social class and other factors, and it has been widely used in educational psychology. Therefore, this study explored whether Mindset can moderate depressive and anxiety symptoms caused by the student-teacher relationship. We have made further revisions to the introduction section.

**Q6: The literature review should not merely list studies or evidence but should critically analyze the literature. Without this, the innovation and necessity of the study are questionable.**

Thank you for your suggestion. We have added some content to provide a more detailed introduction to the more relevant literature, including the research sample and detailed conclusions, as well as an analysis of the shortcomings of previous studies.

**Q7: The literature review on the "The moderating role of mindset" has critical flaws, leading to logical confusion. First, the current literature reasoning does not sufficiently support H3 and H4; the reasoning suggests that "mindset" might function more as a mediator. Second, "mindset" and "growth mindset" are distinct concepts and variables. The authors should confirm and unify the variables and hypotheses (ensuring consistency in the title, hypotheses, and measurement tools). I recommend referencing high-quality core literature in the field as a logical paradigm.**

Thank you for your suggestion. We have now improved this section. We cite and describe in detail the research conducted in recent years on the moderating effect of mindset in mental health to strengthen the basis of the hypothesis. At the same time, regarding the relationship between mindset and growth mindset, we cite Carol's research at the beginning of this section and explain that Carol distinguishes between a fixed mindset and a growth mindset to avoid possible confusion.

**Q8: The literature review lacks sufficient support from recent studies (within the last five years).**

Thank you for your suggestion. We admit that we have previously cited too few recent studies. In this revision, we have added more studies from the past five years and discussed them in more detail.

Research Methods
**Q9: Please provide a detailed description of the sampling process. For example, how were the questionnaires distributed? How was bias controlled during the distribution process? What was the number of valid questionnaire? What were the inclusion and exclusion criteria for valid questionnaire? Was the minimum sample size sufficient, and what is the theoretical basis for it?
For guidance on bias control, please refer to: Podsakoff, P. M., MacKenzie, S. B., Lee, J. Y., & Podsakoff, N. P. (2003). Common method biases in behavioral research: a critical review of the literature and recommended remedies. Journal of Applied Psychology, 88(5), 879.** [**https://doi.org/10.1037/0021-9010.88.5.879**](https://urldefense.proofpoint.com/v2/url?u=https-3A__doi.org_10.1037_0021-2D9010.88.5.879&d=DwQGaQ&c=slrrB7dE8n7gBJbeO0g-IQ&r=7lapq_ae5nufOEz9sHrMRw&m=Nx6xT2p3gZDTVahmQ7MNoLiV1sREeWznn_og_tbwJCvLDqdaHBxgbjsJw3RzQRcK&s=07RMiLHw6MfeDp8RICHVOQhLEu5XJiZ2FwdJZ46Ncd0&e=) **(This is optional should only be taken as a suggestion for the improvement of the manuscript).** 
Thank you for your question about common method biases. In this revision, we enhanced the description of the sampling process and bias control measures to provide a more comprehensive response to the editor’s suggestions. First, we clarified the specific channels through which the survey was distributed, including email and social media. We also elaborated on the screening and bias control criteria, such as excluding duplicate responses, questionnaires with abnormal completion times, and those with more than one-third missing data. Additionally, we emphasized that the survey was conducted anonymously to minimize common method bias, referencing the recommendations of Podsakoff et al. (2003) on reducing biases related to social desirability. Finally, we acknowledged the limitations of relying solely on these methods to eliminate common method bias and discussed future suggestions for further reduction of such biases.

"The questionnaire was collected using the Wenjuanxing platform, participants were invited to complete the survey through electronic links distributed via email and social media. Responses that were incomplete or exhibited patterns of response bias (e.g., completion times under 5 minutes or over 10 minutes) were excluded from analysis. Only fully completed questionnaires with valid data were retained, and all responses recorded were used for analysis. Responses were collected anonymously to reduce common method bias (Podsakoff et al., 2003)."

**Q10: Regarding the measurement tools, I have concerns about the "Mentoring Functions Questionnaire-9 (MFQ-9)" and the "Mindset Scale." Can these tools accurately measure "supervisor-student relationship" and "growth mindset"? Additionally, what is the reliability of these tools?**

Thank you for your suggestions. These two scales have been validated as valid instruments in other studies. For example, Hu et al. (2014) measured the quality of supervisor-student relationships among Taiwanese students using the MFQ-9 and obtained a reliability of 0.93; in addition to this, Midkiff et al. (2014) analyzed the mindset scale using Item Response Theory (IRT), with a Cronbach's α = 0.93. Therefore, we believe that these two scales are appropriate measures of the mentor-student relationship as well as the growth mindset. We have used these data to update the relevant indicators in the article and have indicated the cited literature.

**Q11: The section on ethical approval should be moved before the description of the “Participants and settings”.**

Thank you for your suggestion, we have moved this section to the appropriate section.

Results
**Q12: How many questionnaires were used for the confirmatory factor analysis (CFA)? Note that, methodologically, CFA should not use the same data as exploratory factor analysis (i.e., pilot study).**

Thank you for your analysis. In this study, we first recruited a group of participants (*n* = 325) to complete the pilot study and used these data to perform a CFA. After verifying the reliability of the scale, we recruited a larger sample of participants (*n* = 2,278) to complete the data collection and analysis in the formal study. Therefore, the two parts did not use the same participants.

**Q13: In addition to model fit indices (Table 1), what are the other structural validity indicators (e.g., convergent validity, discriminant validity)?**

Thank you for your suggestions. We did not report all indicators in full in our initial manuscript submission. In the current version, we first reported in the text that all items had factor loadings > 0.7, and computed Average Variance Extracted (AVE) and Composite Reliability (CR) values for each scale from the factor loadings in each scale and added them to Table 1. AVE and CR are indicators of the consistency of the question items within the scale. All of the scales used in this study have a CR > 0.7 and an AVE > 0.5, which provides good reliability. For discriminant validity, we calculated the heterotrait-monotrait ratio (HTMT) between the scales and added it to Table 2. The results showed that the HTMT between all the scales was < 0.85, indicating good discriminant validity between the scales. The details were added to Table 1 and corresponding descriptions.

**Q14: Please provide literature support for the standard value ranges of all indicators.**

Thank you for your advice. We have detailed the range and significance of each indicator in the footnotes to Table 1, citing the reference (Steiger, 1990). We have also provided more detailed description of the scoring method and meaning of each scale.

**Q15: Validity tests could be moved to the methods section and integrated with the measurement tools. The common method bias test should be the first part of the results section and should be moved accordingly.**

Thank you for your advice. Because the pilot study was analysis of the reliability of the scale and was not related to the main purpose of this study, we agree with your suggestion to move it to the methods section. We have now revised this section.

**Q16: Since the accuracy of two scales in measuring the study variables is debatable, the validity of the results is questionable.**

Thank you for your Comment. Given that other studies have also used the MFQ-9 as well as Mindset scales to measure the corresponding psychological traits, we believe that the reliability of these two scales should be sufficient. However, we still value your comment and therefore we have added to this point in the Discussion section that in the future, consideration should be given to conducting surveys using more widely used scales to better ensure the reliability of the study.

Discussion
**Q17: The discussion should focus on analyzing the reasons behind the findings rather than merely restating the results. The discussion would benefit from more recent literature, especially when analyzing reasons, and should consider the Chinese cultural context.**

Thank you for your suggestion. We have discussed the conclusions of this article in more detail in the discussion section. We explored the importance of the professor-student relationship for Chinese students in the context of Confucian thinking in the Chinese cultural context, and further argued that this importance can exacerbate the impact of the professor-student relationship on the mental health of graduate students.

China's educational culture, deeply rooted in Confucian traditions, emphasizes reverence for teachers and scholarly authority. Cross-cultural studies reveal significant variations in how "respect for educators" manifests across societies(e.g. Li, 2006; Li & Fischer, 2007). In Western individualistic contexts (e.g., the United States), student-teacher relationships are typically grounded in reciprocal equality, characterized by what scholars term "horizontal respect" (Renger et al., 2017). Conversely, Confucian-influenced societies (e.g., Taiwan) maintain hierarchical academic relationships where students in junior positions were expected to demonstrate deferential respect, a distinction identified as the most pronounced cross-cultural variation in educational interactions (Spencer-Oatey, 1997). This cultural framework amplifies the psychological significance of supervisor-student relationships for Chinese graduate students. Empirical evidence suggests that relationship quality exerts greater impact on students in Confucian contexts compared to their Western counterparts, with Chinese students more likely to internalize supervisors' evaluations as reflections of personal competence. A survey of the supervisor-student relationship was conducted among students in China and the UK by Spencer-Oatey (1997). The results showed that Chinese students felt a greater power gap with their mentors, thereby impacting their research self-efficacy. This pattern was also found in the following analyses in the current study.

**Q18: What are the practical implications of this study? The authors should propose actionable recommendations** **based on the findings.**

Thank you for your suggestion. We have now added a section to the significance section with practical and actionable recommendations based on the key findings of this study.

The findings of this study provide practical implications for enhancing the mental health of Chinese graduate students. First, universities should systematically establish a mentorship quality monitoring system by integrating supervisor-student relationship management into supervisor training programs. Evidence-based courses on mentoring strategies should be developed, as existing research demonstrates that supervisors' instrumental support (e.g., academic guidance) and emotional support positively correlate with students' subjective well-being (L. Ma et al., 2024). Therefore, cultivating supervisors’ emotional intelligence and autonomy-granting techniques should be prioritized. Second, graduate training programs need to incorporate psychological capital development components. Grounded in Self-Determination Theory – which identifies autonomy, competence, and relatedness as three fundamental psychological needs – future interventions should design self-efficacy enhancement modules through structured training featuring phased goal achievement and peer mentoring exchanges. Third, universities should innovate mindset intervention models by embedding growth mindset cultivation into academic supervision. Aronson et al. (2002) demonstrated that simple interventions like lectures and video observations significantly improve students' growth mindset, a finding corroborated by Andersen & Nielsen's(2016) study showing educational pamphlets and videos effectively enhance children's malleable thinking. Implementing these systemic measures requires coordinated efforts among academic affairs departments, faculty teams, and student support services, supported by continuous outcome evaluation to establish dynamic optimization mechanisms, ultimately forming an ecosystem conducive to graduate students' mental health development.

Limitations
**Q19: Please suggest specific solutions to address the study’s limitations rather than general statements. For example, what objective measurement methods could future studies use to assess variables like anxiety?**

Thank you for your suggestion. We have now rewritten the limitation section to provide specific solutions to the specific problems raised in this study and to cite references.

"This study employed a questionnaire survey to collect data from graduate students across different academic levels in China. However, the research has several limitations: First, while cross-sectional data analysis revealed significant correlations among the key variables, the cross-sectional design precludes causal inference (Mitchell & and Maxwell, 2013; Wunsch et al., 2010). To explore the potential causal mechanisms underlying the relationship between supervisor-student relationships and anxiety/depression symptoms, future studies should adopt longitudinal designs with multi-wave data collection for predictive analysis. Second, although anonymous questionnaire administration was implemented to mitigate social desirability bias, self-reported measurements may still entail common method bias. The dual-control strategies proposed by Podsakoff et al. (2003) offer solutions: procedurally through psychologically separated questionnaire designs (e.g., interleaving items from distinct research topics), and statistically via Multitrait-Multimethod (MTMM) models to disentangle method variance. Third, while focusing on generalized anxiety, existing evidence demonstrates significant associations between self-efficacy and specific anxiety subtypes such as social anxiety (Iancu et al., 2015; Lipp et al., 2023). Future investigations should differentiate anxiety manifestations in graduate populations (e.g., academic anxiety, career development anxiety) to develop targeted interventions. Additionally, incorporating validated psychological mediators like subjective well-being and resilience would help systematically construct theoretical models of how supervisory support influences graduate mental health."

In summary, the manuscript requires substantial revisions before it can be considered for publication. I look forward to reviewing an improved version of your work.

6. PLOS authors have the option to publish the peer review history of their article ([what does this mean?](https://urldefense.proofpoint.com/v2/url?u=https-3A__track.editorialmanager.com_CL0_https-3A-252F-252Fjournals.plos.org-252Fplosone-252Fs-252Feditorial-2Dand-2Dpeer-2Dreview-2Dprocess-2523loc-2Dpeer-2Dreview-2Dhistory_1_010f0195b9988848-2D966fb460-2D2170-2D4ca3-2D9ad4-2D3374b9636348-2D000000_mnAoJ-2Dw4WBtjxq9r3izGlmm-5FnZE-2Dsh0xZ5kWeNoJXj0-3D203&d=DwMGaQ&c=slrrB7dE8n7gBJbeO0g-IQ&r=7lapq_ae5nufOEz9sHrMRw&m=Nx6xT2p3gZDTVahmQ7MNoLiV1sREeWznn_og_tbwJCvLDqdaHBxgbjsJw3RzQRcK&s=lkZfV2zs5Slt2srkGsqySj4BWq4R03NNWmBDZzDO53M&e=)). If published, this will include your full peer review and any attached files.

If you choose “no”, your identity will remain anonymous but your review may still be made public.

**Do you want your identity to be public for this peer review?** For information about this choice, including consent withdrawal, please see our [Privacy Policy](https://urldefense.proofpoint.com/v2/url?u=https-3A__track.editorialmanager.com_CL0_https-3A-252F-252Fwww.plos.org-252Fprivacy-2Dpolicy_1_010f0195b9988848-2D966fb460-2D2170-2D4ca3-2D9ad4-2D3374b9636348-2D000000_DHFvZx7zPnMHsQn2y324FFb5Dh7-2DWE0Ms2m7oe2HcdE-3D203&d=DwMGaQ&c=slrrB7dE8n7gBJbeO0g-IQ&r=7lapq_ae5nufOEz9sHrMRw&m=Nx6xT2p3gZDTVahmQ7MNoLiV1sREeWznn_og_tbwJCvLDqdaHBxgbjsJw3RzQRcK&s=HawVePXr9li7wDNohbGQHQXCXDxyagtDUAoBcqtefJ4&e=).

Reviewer #1: No
